# Interpretation of A1C measurement in sub-Saharan Africa beyond the global A1C-Derived Average Glucose (ADAG) equation

**DOI:** 10.11604/pamj.2023.44.8.32984

**Published:** 2023-01-05

**Authors:** Eric Balti, Chris Nadege Nganou-Gnindjio, Brice Enid Nouthe, Valery Effoe, Valentin Siaha, Mesmin Dehayem, Eugene Sobngwi, Jean-Claude Mbanya

**Affiliations:** 1 Endocrine and Diabetes Unit, National Center of Obesity, Yaounde Central Hospital, Yaounde, Cameroon,; 2 Department of Internal Medicine, Faculty of Medicine and Pharmaceutical Sciences, University of Dschang, Dschang, Cameroon,; 3 Department of Internal Medicine and Diabetes Research Center, Vrije Universiteit Brussel - VUB, Brussels, Belgium,; 4 Internal Medicine and Specialities Department, Faculty of Medicine and Biomedical Sciences, University of Yaoundé I, Yaoundé, Cameroon,; 5 Fraser Health Authority/Department of Medicine, University of British Columbia, Vancouver, Canada,; 6 Department of Cardiology, Morehouse School of Medicine, Atlanta, GA 30310, United States of America,; 7 Laboratory of Molecular Medicine and Metabolism, Biotechnology Centre, Nkolbisson, University of Yaoundé I, Yaoundé, Cameroon

**Keywords:** Blood glucose, hemoglobin A, diabetes mellitus, glucose monitoring

## Abstract

**Introduction:**

optimal metabolic control is crucial for prevention of diabetes associated complications. HbA1c is a correlate of chronic hyperglycemia and is associated with long-term diabetes complications. We investigate the relationship between A1C and estimated average blood glucose (eAG) from the multicenter A1C-Derived Average Glucose (ADAG) study, in a sub-Saharan African population.

**Methods:**

forty-seven patients with diabetes mellitus and ten normoglycemic individuals were consecutively recruited from a tertiary reference hospital in Cameroon. This observational study was conducted in the framework of the ADAG study. eAG was derived from single values obtained from self-monitored blood glucose (SMBG) and from continuous glucose monitoring (CGM). Spearman correlation coefficient was used to examine the relationship between eAG and A1C levels.

**Results:**

there was a strong linear relationship between eAG using SMBG with A1C level; eAG (mmol/l) =1.22 x A1C (%) - 0.25; R2 = 0.58; p<0.001. This suggests that a one percent increase in A1C corresponds to a 1.22 mmol/l increment of eAG. A similar relationship was found between A1C level and eAG from the continuous glucose monitoring (CGM) measurements albeit with a smaller accretion; eAG (mmol/l) =0.95 x A1C (%) + 1.52; R2 = 0.52; p<0.001. The bias of the global ADAG equation was lower than 5% below A1C level of 7% and progressively increased with higher values of A1C.

**Conclusion:**

consistent with previous reports, using a population specific equation, A1C can be better derived from eAG in individuals from sub-Saharan African origin.

## Introduction

Glycosylated hemoglobin (A1C), the stable glucose adduct to the N-terminal group of the beta-chain of HbA0, is commonly used for the long-term control of blood glucose (BG) in patients with diabetes mellitus [1]. This is the mainstay for the evaluation of diabetes management together with the analysis of glycaemia from frequent blood samples and when possible the continuous glucose monitoring (CGM) [2]. Glycosylated hemoglobin is now a consensual accepted parameter for the diagnosis of diabetes mellitus. As opposed to single measures of glucose concentration, it is a marker for the presence of diabetes but also a surrogate of its complications [3]. In addition, using multiple blood glucose from samples of the preceding days and months, several reports demonstrated a strong correlation between A1C and mean BG levels [4-6], and therefore confirmed A1C as an estimate of mean BG evaluation.

Evidence from large cohort studies and clinical trials such as the Diabetes Control and Complications Trial (DCCT) [7], the United Kingdom Prospective Diabetes Study (UKPDS) [8], the Action in Diabetes and Vascular Disease: Preterax and Diamicron MR Controlled Evaluation (ADVANCE) trial [9], the Action to Control Cardiovascular Risk in Diabetes (ACCORD) trial, the Veterans Affairs Diabetes Trial (VADT), and the Hyperglycemia and Its Effect After Acute Myocardial Infarction on Cardiovascular Outcomes in Patients With Type 2 Diabetes Mellitus (HEART2D) trial [10] suggest that improved glycemic control, when instigated early, has the potential to tremendously reduce the risk of micro- and macrovascular complications. On the other hand, currently available data suggest that populations of African descent with diabetes have a decreased accessibility to care and a higher prevalence of microvascular complications together with amputations [7,11-13].It is therefore critical to find accurate surrogates of glucose control for optimal care and patients´ follow up. Between the estimated average glycaemia and A1C level, a linear regression equation (estimated average BG (eAG)_mmol/l_ = 1.59 x A1C - 2.59 (eAG_(mg/dl_) = 28.7 x A1c - 46.7), has been established by the A1C-Derived Average Glucose (ADAG) Study Group [5]. Because this equation represents an overall relationship, and differences in A1C levels exist between populations of different origins [1,14-16], we sought to investigate, in a sub-Saharan African population, the relationship between A1C and eAG in order to translate it into a standardized parameter for both healthcare practioners and patients. Our pre-specified hypothesis was that a population specific aAG formula would need to be developed and used in our context.

## Methods

**Study design:** this was an observational study conducted in the framework of the A1C-Derived Average Glucose (ADAG) study, fully described elsewhere [5]. The study complies with the STROBE guidelines.

**Study setting and population:** from April 2006 to December 2007, we consecutively enrolled 57 patients and controls aged between 18-69 years old at the National Obesity Centre of Yaounde Central Hospital, Cameroon. Briefly, each normal control and patient with type 1 or type 2 diabetes was enrolled after stable glycemic control was ascertained by an A1C variation of less than one percentage unit within the six months prior to enrolment. Diabetes was ruled out by a blood glucose level lower than 5.4 mmol/l after an overnight fast and an A1C less than 6.5%. Seventy persons were volunteers for the study; among those, 13 (18.5%) were excluded because of presence of hemoglobin AS trait.

**Variables:** the main variables of interest included age, gender, type of diabetes mellitus, body mass index, CGM-derived glucose values, self-monitored blood glucose (SMBG)-derived glucose values and eAG as well as HbA1c.

### Data resource and measurement

Data collection and collection tools: study participants underwent one visit every four weeks and were asked to report SMBG by fingerstick and to use the CGM (Medtronic Minimed, Northridge, CA, USA) during at least 48 hours at baseline. Glucose levels in the extracellular fluid of the abdominal subcutaneous tissue were monitored every five minutes during the CGM and participants performed an eight-point profile of SMBG (preprandial, 90 min postprandial, bedtime and 03:00 hours). During the three weeks when CGM was not done, participants performed a seven-point SMBG (preprandial, postprandial and bedtime) for at least two week days and one day in the week-end. Hemocue Glucose 201 Plus meter (Hemocue, Angelholm, Sweden) was used for the eight-point SMBG while OneTouch Ultra glucose monitoring devices (Lifescan, Milpitas, CA, USA) were used for the seven-point SMBG. At the last visit, only the seven-point SMBG was performed. Fasting BG was considered to be the pre-breakfast BG and pre- and post-prandial measurements from SMBG (Hemocue) were used to calculate mean pre- and post-prandial BG, as well as pre- and post-breakfast, lunch and dinner values. The measurement of A1C was done at the National Obesity Centre using the DCCT-aligned machine DCA 2000®+, Bayer Healthcare LLC, inc. Elkhart, IN 46514 USA. For the second measurement, frozen samples were shipped overnight on dry ice to the European Reference Laboratory for A1C at Zwolle, Netherlands. A1C values retained were the mean of 4 measurements obtained from different DCCT-aligned assays, including a high-performance liquid chromatography assay (Tosoh G7; Tosoh Bioscience, Tokyo, Japan), two immunoassays (Roche A1C and Roche Tina-quant; Roche Diagnostics), and an affinity assay (Primus Ultra-2; Primus Diagnostics, Kansas City, MO) all approved by the National Glycohemoglobin Standardization Program (NGSP) as previously reported [17].

For optimal accuracy, the initial two hours of CGM (calibration period) were excluded and CGM results were only used if at least one successful 24-h profile out of the 2-3 days of monitoring was available with no gaps >120 min and a mean absolute difference compared with the Hemocue calibration results <18% [7]. Those results were pooled with specific SMBG results to calculate the estimate of eAG concentration.

**Sample size:** study participants were consecutively included and a convenience sample was used for the purpose of the study.

**Data analysis:** statistical analyses were done using SPSS for Windows software version 22.0 (SPSS, Chicago, IL). Results are expressed as median (interquartile range) or number of observation (percentage). Test of normality was performed using Kolmogorov-Smirnov test. Continuous variables were compared by Kruskal-Wallis one-way analysis of variance or Mann-Whitney U test and categorical variables by Chi-square test with Yates´ continuity correction or Fischer´s exact test. Correlations between variables were evaluated using both Spearman's rank test using a complete case analysis. The bias obtained from the use of the global ADAG equation was calculated as the percentage of difference between the population specific eAG and the result obtained with the ADAG equation. The cutoff for significance was p value < 0.05.

**Ethical consideration:** the study was approved by the National Ethics Committee of Cameroon and all study participants or their legal representative provided informed consent.

## Results

**Sociodemographic analysis:** among the 57 study participants, 10 (17.5%) were non-diabetic volunteers and among patients with diabetes 20 (42.6%) - of which 6 (12.8%) were patients with T2D - were treated with insulin. The overall sex-ratio male/female was slightly lower than 2: 1. Table 1 summarizes the characteristics of the study population.

**Table 1:** baseline characteristics of the study population

Parameters	Type 1 diabetes (n = 14)	Type 2 diabetes (n=33)	Healthy volunteers (n=10)	p value^a^
Age (years)	23 (20-34) ^b^	48 (41-52) ^c^	24 (23-25)	<0.001
Sex (M/F)	8/6	21/12	7/3	0.865
BMI at entry	22 (21-23) ^b^	27 (24-30) ^c^	22 (21-24)	<0.001
SBP, mmHg	122 (116-128)	127 (116-135)	122 (114-126)	0.287
DBP, mmHg	80 (71-84)	82 (74-89)	72 (66-78)	0.055
A1C (%)	9.3 (7.2-11.0) ^d, e^	6.7 (5.8-7.4) ^e^	4.9 (4.7-5.0)	0.001
Insulin treated, n (%)	14 (100)	8 (24)	na	<0.001
CGM-derived eAG, mmol/l	10.0 (8.2-12.7) ^b, c^	7.5 (6.0-9.0) ^c^	5.5 (5.3-5.9)	<0.001
mg/dl	180 (148-229) ^b, c^	134 (112-162) ^c^	99 (95-105)	<0.001
SMBG-derived eAG, mmol/l	10.1 (8.2-13.0) ^b, c^	7.5 (6.7-9.0) ^c^	5.5 (5.4-5.7)	<0.001
mg/dl	182 (147-234) ^b, c^	136 (121-163) ^c^	99 (98-102)	<0.001

Values are median (interquartile range) or count (percentage); ^a^: overall p-value; ^b^: p <0.001 vs type 2 diabetes; ^c^: p <0.001 vs healthy volunteers; ^d^: p <0.05 vs type 2 diabetes; ^e^: p <0.05 vs healthy volunteers; na: not applicable; BMI: body mass index; SBP: systolic blood pressure; CGM: continuous glucose monitoring; SMBG: self-monitored blood glucose; eAG: estimated average blood glucose

**Figure 1:**
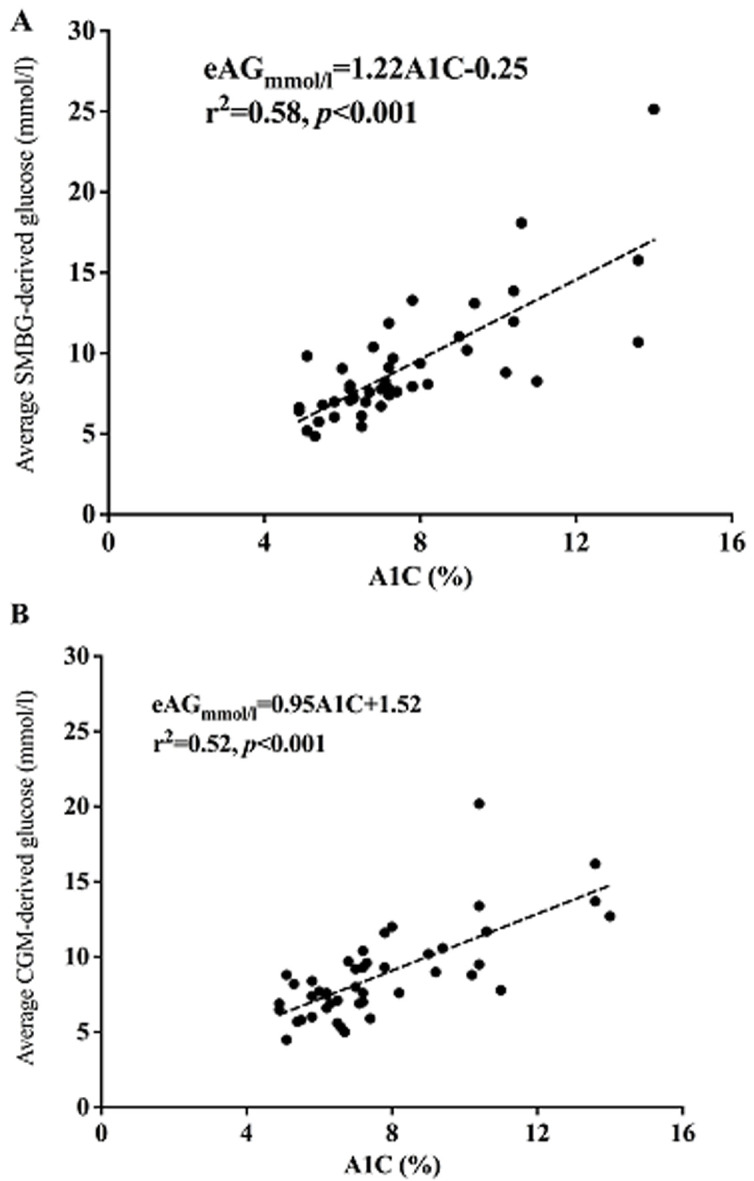
A) scatter plot diagrams (linear relationship) of average glucose values derived from self-monitored blood glucose (SMBG); and B) continuous glucose monitoring (CGM) against plasma A1C values.

**Association study:** both eAG using seven-point SMBG and CGM were correlated with A1C (Figure 1 (A,B)). The linear regression could be defined by the following regression equation: eAG (mmol/l) =1.22 x A1C (%) - 0.25, r= 0.58 [eAG (mg/dl) =21.98 x A1C (%) - 4.50] for capillary BG during SMBG. From this equation, it can be estimated that per 1% increase of A1C, the mean increase in average BG is 1.22 mmol/l. Using CGM (interstitial), a linear regression was also found and the following equation between average BG and A1C was obtained eAG_(mmol/l)_= 0.95 x A1C _(%)_+ 1.52, r=0.52 (eAG_(mg/dl)_= 17.1 x A1C_(%)_+ 27.4). When using the standardizing formula from the National Glycohemoglobin Standardization Program (NGSP) to International Federation of Clinical Chemistry and Laboratory Medicine (IFCC), NGSP unit = (0.915 x IFCC) + 2.15 [18], we were able to estimate that a 1 mmol/mol increase in A1C (in IFCC unit) using SMBG corresponds to 0.87 mmol/l (16 mg/dl) increase in eAG.

**Comparison with the global ADAG equation:** the use of the ADAG equation induced a bias that was lower than 5% for an A1C value of ≤ 7% for CGM-derived eAG. However, the deviation from point-of-care (One Touch) derived eAG was lower, with a bias below 5% for an A1C level of ≤ 7.5%. Nevertheless, in both cases the discrepancy with eAG obtained from the overall ADAG equation increased gradually with A1C level until a bias greater than 10% was obtained for A1C values above 9.5% in the case of measurement using One Touch apparatus (Table 2).

**Table 2:** estimated average blood glucose (eAG) values using the overall ADAG formula and their corresponding levels from the Cameroonian population-derived equations; the deviations (biases) from the continuous glucose monitoring (CGM)- and self-monitored blood glucose (SMBG)-derived measurements are expressed in percentage

NGSP HbA1c (%)	Estimated average glucose (eAG, mmol/l)			Bias	
	From the overall ADAG equation	From the Cameroonian sub-population		CGM-bias (%)	SMBG-bias (%)
		CGM	SMBG		
6.0	6.95	7.22	7.07	-3.74	-1.70
6.5	7.75	7.70	7.68	0.65	0.85
7.0	8.54	8.17	8.29	4.53	3.02
7.5	9.34	8.65	8.9	7.98	4.89
8.0	10.13	9.12	9.51	11.07	6.52
8.5	10.93	9.60	10.12	13.86	7.95
9.0	11.72	10.07	10.73	16.39	9.23
9.5	12.52	10.55	11.34	18.68	10.36
10.0	13.31	11.02	11.95	20.78	11.38
10.5	14.11	11.50	12.56	22.71	12.30
11.0	14.90	11.97	13.17	24.48	13.14
11.5	15.70	12.45	13.78	26.11	13.90
12.0	16.49	12.92	14.39	27.63	14.59

eAG: estimated average blood glucose; NGSP: National Glycohemoglobin Standardization Program; CGM: continuous glucose monitoring; SMBG: self-monitored blood glucose; CGM-bias: bias of the overall ADAG equation compared to the equation derived from CGM measurements in the Cameroonian population; SMBG-bias: bias of the overall ADAG equation compared to the equation derived from SMBG measurements in the Cameroonian population

**Funding:** this work was realized in framework of the ADAG study and was supported by research grants from the American Diabetes Association and European Association for the Study of Diabetes. Additional funding was received from Abbott Diabetes Care, Bayer Healthcare, GlaxoSmithKline, Sanofi-Aventis Netherlands, Merck, Lifescan, and Medtronic Minimed. Supplies and equipment were provided by Medtronic Minimed, Lifescan, and Hemocue.

## Discussion

Using SMBG and CGM, this study has demonstrated a linear relationship between eAG and A1C in a population of sub-Saharan Africans. According to the linear equation obtained, a mean increase of eAG per 1% increment in A1C is 1.22 mmol/l. This is relatively low compared to that observed in DCCT study of type 1 diabetic patients only (2.0 mmol/l) [19]. However, this approximates the result obtained from the ADAG study (1.59 mmol/l) albeit the lower magnitude.

We describe the relationship between A1C and eAG in a sub-Saharan setting where the highest increase in diabetes incidence is predicted in this part of the word facing drastic scarcity of resources. This challenge relates not only to the management of current cases of the disease but also for the expected new cases in the coming years [20]. It is therefore important to find cost-effective alternative solutions to accurately assess diabetes control and therefore delay the onset of diabetes-associated chronic complications [21]. Current data from meta-analyses suggest a net difference in A1C levels between people of African descent and non-Hispanic Whites with a reported 0.65% difference in A1C levels between the two populations [15]. Nevertheless, this difference does not preclude the use of A1C in the follow up of people from sub-Saharan Africa since our analysis advocate a significant correlation between A1C and eAG. In comparison to the global ADAG equation, it is likely that there is a good relationship until a certain value of A1C above which the magnitude of the bias increases with A1C values. Indeed, patients with T1D had a significantly higher A1C than those with T2D and healthy controls. One could therefore speculate that the overall ADAG equation could better apply to the latter two groups than the former. This is however true in all patients with diabetes mellitus and uncontrolled disease. Our observations need however to be confirmed in a larger population and the appropriate cutoff value for the concordance between equations warrants further investigation.

Our study has the advantage to reveal a linear relationship between A1C and eAG in a heterogeneous sample of non-diabetic healthy volunteers and patients with both type 1 and 2 diabetes. The limitations of our study include the small sample size which did not allow us to conduct subgroup analysis. The exclusion of subjects with erythrocyte disorders restricted our equation to 60-98% of the sub-Saharan African population [22]. This suggests that another equation or the use of another proxy of glucose control might be needed in this population. Also, all patients with diabetes were regularly followed up while this is not the case for many patients in resource limited areas. This might therefore impact the external validity of our findings [23]. Nevertheless, at the time of the study, most patients (61.1% of the participants) had an A1C level above 8% despite the regular follow up; which reproduces the average condition of patients in our setting.

## Conclusion

We have shown that in a population of healthy volunteers and patients with diabetes mellitus, A1C is strongly correlated to eAG which can easily be derived using a population specific formula. The current global ADAG equation can be used up to a threshold A1C above which the magnitude of the associated bias is increased.

### What is known about this topic

- *A1C is a surrogate marker of glycemic control and can be derived from blood glucose levels;*
- *It represents a valuable tool for treatment optimization as well as prevention of diabetes associated complications*.

### What this study adds

- *The relationship between A1C and blood glucose can be defined by a population specific equation in sub-Saharan populations;*
- *The global ADAG derived A1C could be used in this population up to a threshold value of A1C (7.5% and 7.0% compared to SMBG and CGM, respectively) above which the magnitude of the associated bias is increased*.
